# *In vivo* labeling of endogenous genomic loci in *C. elegans* using CRISPR/dCas9

**DOI:** 10.17912/micropub.biology.000701

**Published:** 2022-12-13

**Authors:** Nadin Memar, Aditya Sethi, Sebastian Luehr, Eric J Lambie, Barbara Conradt

**Affiliations:** 1 Institute for Basic Science (IBS), Ulsan 44919, South Korea; 2 University College London, United Kingdom; 3 Ludwig-Maximilians-University Munich, Germany

## Abstract

Visualization of genomic loci with open chromatin state has been reported in mammalian tissue culture cells using a CRISPR/Cas9-based system that utilizes an EGFP-tagged endonuclease-deficient Cas9 protein (dCas9::EGFP) (Chen et al. 2013). Here, we adapted this approach for use in *Caenorhabditis elegans* . We generated a *C. elegans* strain that expresses the dCas9 protein fused to two nuclear-localized EGFP molecules (dCas9::NLS::2xEGFP::NLS) in an inducible manner. Using this strain, we report the visualization in live *C. elegans* embryos of two endogenous repetitive loci, *rrn-4* and *rrn-1* , from which 5S and 18S ribosomal RNAs are constitutively generated.

**Figure 1. In vivo labeling of rrn-4 and rrn-1 genomic loci using dCas9::NLS::2xEGFP::NLS.**
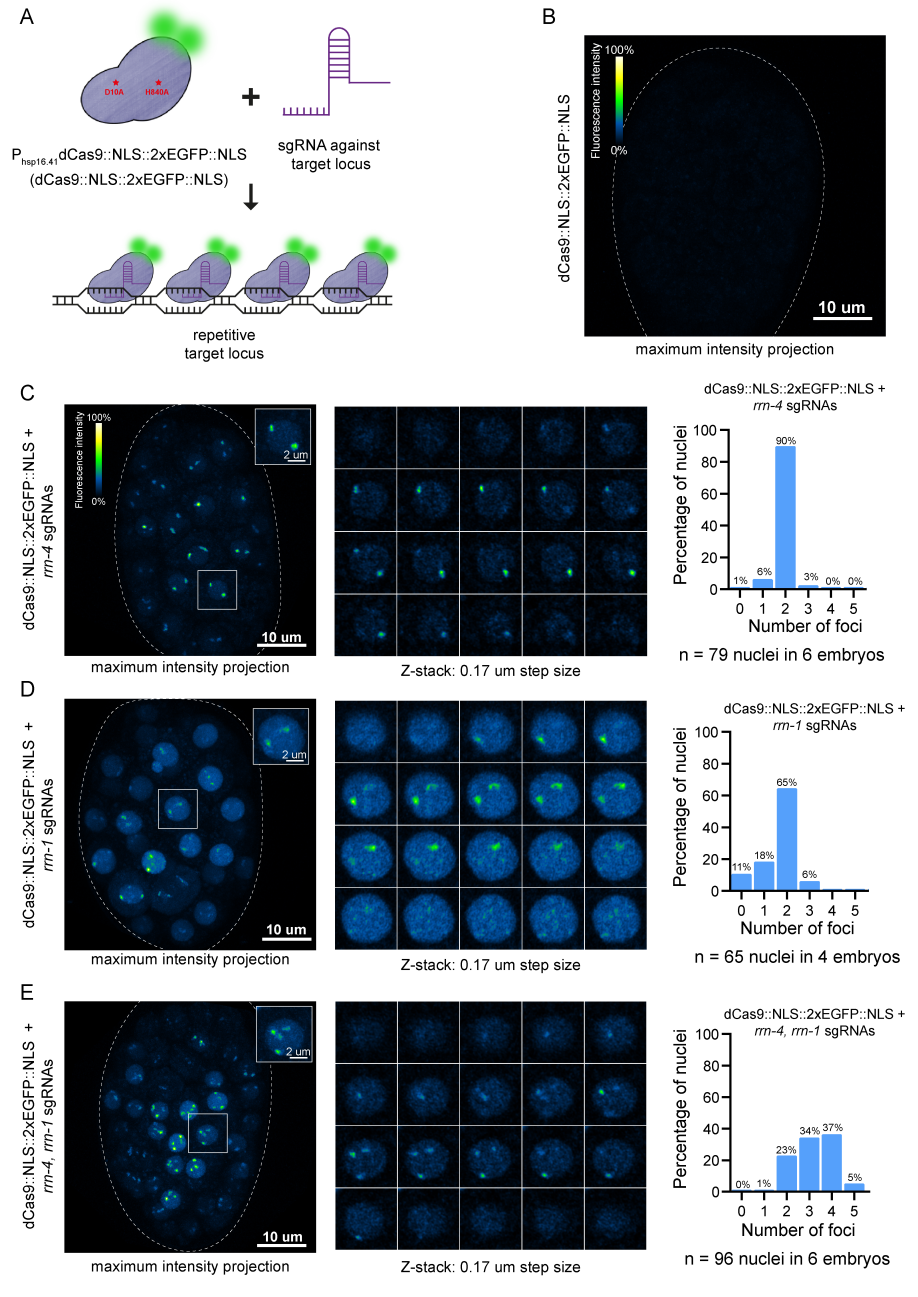
( **A** ) Schematic representation for the labeling of genomic loci using the dCas9::NLS::2xEGFP::NLS system. Red stars indicate the amino acid changes in Cas9 to make it nuclease-deficient. ( **B** ) Maximum intensity projection of a representative embryo expressing *dCas9::NLS::2xEGFP::NLS* without sgRNAs. In total, we looked at 7 embryos. The white dotted line marks the eggshell of the embryo. Fluorescence intensity panel: 0% = 0 pixels, 100% = 1925 pixels. ( **C, left panel** ) Maximum intensity projection of a representative embryo expressing *dCas9::NLS::2xEGFP::NLS* with 4 sgRNAs against the *rrn-4* target locus. The white dotted line marks the eggshell of the embryo. The white box highlights a representative nucleus with two foci. Fluorescence intensity panel: 0% = 0 pixels, 100% = 1925 pixels ( **C, middle panel** ) The Z-stack (step size = 0.17µm) of the highlighted nucleus with the two foci is shown. ( **C, right panel** ) Quantification of the number of foci per nucleus in 6 embryos is shown. The percentage is indicated on top. ( **D, left panel** ) Maximum intensity projection of a representative embryo expressing *dCas9::NLS::2xEGFP::NLS* with 4 sgRNAs against the *rrn-1* target locus. The white dotted line marks the eggshell of the embryo. The white box highlights a representative nucleus with two foci. Fluorescence intensity panel: 0% = 0 pixels, 100% = 1925 pixels ( **D, middle panel** ) The Z-stack (step size = 0.17µm) of the highlighted nucleus with the two foci is shown. ( **D, right panel** ) Quantification of the number of foci per nucleus in 4 embryos is shown. The percentage is indicated on top. ( **E, left panel** ) Maximum intensity projection of a representative embryo expressing *dCas9::NLS::2xEGFP::NLS* with 4 sgRNAs against each *rrn-4* and *rrn-1* target loci. The white dotted line marks the eggshell of the embryo. The white box highlights a representative nucleus with four foci. Fluorescence intensity panel: 0% = 0 pixels, 100% = 1925 pixels ( **E, middle panel** ) The Z-stack (step size = 0.17µm) of the highlighted nucleus with the four foci is shown. ( **E, right panel** ) Quantification of the number of foci per nucleus in 6 embryos is shown. The percentage is indicated on top.

## Description

CRISPR/Cas9 has proven to be a revolutionary tool in the life sciences (Adli 2018), and extensive efforts have been made to optimize this system for genome editing in various model organisms (Wang et al. 2016, Farboud 2017, Lee et al. 2020, Sharma et al. 2021, Zirin et al. 2022, Son et al. 2022). In addition, an endonuclease-deficient mutant version of *Streptococcus pyogenes* Cas9 (dCas9) was developed for the purpose of visualizing specific endogenous loci within the genome. This mutant version was generated by introducing mutations that result in two amino acid changes in the Cas9 protein (D10A and H840A) (Gilbert et al. 2013, Qi et al. 2013). The resulting dCas9 protein can be targeted to a specific locus by single guide RNAs (sgRNAs) but binding of the sgRNA-dCas9 complex to the target locus does not result in target locus cleavage. Furthermore, fusing dCas9 to a fluorescent protein such as EGFP (enhanced GFP) (dCas9::EGFP) allows the labelling of the target locus in living cells. However, it has been proposed that successful binding of the sgRNA-dCas9::EGFP complex (and hence labelling of the target locus) is dependent on the target locus being in an open chromatin state (Verkuijl et al. 2019).

Our aim was to establish a fluorescent CRISPR/dCas9 system for *C. elegans* and to target it to a repetitive endogenous locus (Fig. 1A). To that end, we fused the *Streptococcus pyogenes* *dCas9* coding region to two copies of the *EGFP* coding region, flanked by nuclear localization sequences (NLS). The resulting gene fusion was put under the control of a heat-inducible promoter (P *_hsp-16.41_ dCas9::NLS::2xEGFP::NLS* ). A single copy of the P *_hsp-16.41_ dCas9::NLS::2xEGFP::NLS* transgene was inserted on chromosome II using MosSCI technology (see Methods) (Frokjaer-Jensen et al. 2014). This allowed us to consistently obtain low levels of dCas9::NLS::2xEGFP::NLS protein in an inducible manner in many nuclei throughout the embryo. Ribosomal RNA (rRNA) genes are found in two clusters in the *C. elegans* genome (Spieth et al. 1993, Spieth et al. 2014). The 35S cluster is located at the right end of chromosome I and consists of approximately 55 tandem repeats of a ~7.2 kb region, each containing one copy each of the 18S ( *rrn-1* ), 5.8S ( *rrn-2* ) and 26S ( *rrn-3* ) rRNA genes (Ellis et al. 1986). The 5S cluster is located on chromosome V and consists of approximately 110 tandem repeats of a ~1.0 kb region containing one copy of the 5S rRNA gene ( *rrn-4* ) (Sulston et al. 1974, Nelson et al. 1985). As proof of concept, we targeted dCas9::NLS::2xEGFP::NLS to the *rrn-1* locus and the *rrn-4* locus (Sulston et al. 1974). To that end, we designed four sgRNAs for each of the two loci and, using PCR stitching, fused each set of four sgRNAs to the *C. elegans* U6 promoter to generate P *_U6_ rrn-1(sgRNAs1-4)* and P *_U6_ rrn-4(sgRNAs1-4* ) (Dickinson et al. 2013, Friedland et al. 2013, Ward 2015). The P *_U6_ rrn-1(sgRNAs1-4)* and P *_U6_ rrn-4(sgRNAs1-4* ) transgenes were injected, either separately or in combination, into the *C. elegans* strain containing the integrated transgene P *_hsp-16.41_ dCas9::NLS::2xEGFP::NLS* and transgenic lines harboring extrachromosomal arrays were generated (see Materials and Methods). Expression of P *_hsp-16.41_ dCas9::NLS::2xEGFP::NLS* was induced (heat shock at 28 or 29°C for 30 min) and embryos (25-50-cell stage) were analyzed using a confocal microscope (Zeiss LSM 980 with AiryScan2). In embryos carrying either the P *_U6_ rrn-4(sgRNAs1-4* ) array or the P *_U6_ rrn-1(sgRNAs1-4* ) array, bright dCas9::NLS::2xEGFP::NLS ‘foci’ were detected in nuclei (Fig. 1C, D). In contrast, in control embryos only containing the transgene P *_hsp-16.41_ dCas9:NLS::2xEGFP::NLS* , essentially no nuclear dCas9::NLS::2xEGFP::NLS signal was detected (Fig. 1B). A possible explanation for why we did not detect any signal in the control strain could be that without sgRNAs, dCas9::NLS::2xEGFP::NLS protein cannot bind to DNA and hence exists in an unbound state in the nucleus. Consequently, during nuclear envelop breakdown and cell division, unbound dCas9::NLS::2xEGFP::NLS would diffuse into the cytoplasm resulting in the detection of essentially no signal in the nucleus. In support of this, we observed that in the presence of sgRNAs, dCas9::NLS::2xEGFP::NLS remains bound to DNA during nuclear envelop breakdown and cell division (In Fig. 1D, left panel, a dividing cell can be observed in the bottom half of the embryo). Therefore, the dCas9::NLS::2xEGFP::NLS foci observed are dependent on the presence sgRNAs. Based on these observations, we propose that the foci detected represent the endogenous repetitive *rrn-4* and *rrn-1* loci. Furthermore, the 35S and 5S clusters containing the *rrn-1* or *rrn-4* genes span a ~396kb or ~110kb genomic region, respectively. For this reason, what we refer to as ‘foci’ in this study are actually elliptical and elongated signals that can be detected in more than one Z-slice (for example, see Fig. 1C, D and E, middle panel).

We counted the number of dCas9::NLS::2xEGFP::NLS foci in 79 nuclei from 6 embryos carrying the P *_U6_ rrn-4(sgRNAs1-4* ) array. We found that 90% of these nuclei contained 2 foci, supporting the notion that they represent the endogenous *rrn-4* loci on the two homologous chromosomes V (Fig. 1C). We also detected foci in embryos carrying the P *_U6_ rrn-1(sgRNAs1-4* ) array; however, these foci were less bright than the foci detected in animals carrying the P *_U6_ rrn-4(sgRNAs1-4* ) array (Fig. 1D). We counted 65 nuclei from 4 embryos and found that 65% contained 2 foci, again supporting the notion that these foci represent the endogenous *rrn-1* loci on the two homologous chromosomes I. In addition, we found that 18% of the nuclei had 1 focus, 11% 0 foci and 6% 3 foci (Fig. 1D). Next, we analyzed embryos carrying both the P *_U6_ rrn-1(sgRNAs1-4)* and P *_U6_ rrn-4(sgRNAs1-4* ) array and detected more than 2 foci in the majority of nuclei (Fig. 1E). We counted 96 nuclei from 6 embryos and found that 37% of the nuclei contained 4 foci and 34% contained 3 foci (Fig. 1E). The 4 foci most likely represent the two *rrn-1* loci on the two homologous chromosomes I and the two *rrn-4* loci on the two homologous chromosomes V. Why did we not detect more nuclei with 4 foci? One explanation could be that because of close spatial proximity of foci within the nucleus, we failed to resolve independent foci in those nuclei in which we detected only 2 or 3 foci. Alternatively, the sgRNAs against one of the two loci may be less efficient in targeting dCas9::NLS::2xEGFP::NLS to the respective locus, and this could result in detectable labeling of only two or three rather than four loci. In animals carrying the P *_U6_ rrn-1(sgRNAs1-4)* array targeting the *rrn-1* , foci were less bright than in animals carrying the P *_U6_ rrn-4(sgRNAs1-4)* array targeting the *rrn-4* , and we detected 2 foci in only 65% instead of 90% of the nuclei. Based on these observations, we speculate that binding of *rrn-1(sgRNAs1-4)* to dCas9 protein may be less efficient. Alternatively, the difference may be caused by the fact that the *rrn-1* locus contains 55 copies of the 18S rRNA gene whereas the *rrn-4* locus contains 110 copies of the 5S rRNA gene. It is also possible that the two *rrn-1* loci are less accessible (i.e. more closed chromatin) to the dCas9::NLS::2xEGFP::NLS protein.

In conclusion, we successfully generated a *C. elegans* strain expressing *dCas9* :: *NLS* :: *2xEGFP* :: *NLS* under the control of a heat-inducible promoter. Furthermore, using this inducible dCas9::NLS::2xEGFP::NLS system and appropriate sgRNAs, we show for the first time that it is possible to visualize repetitive endogenous loci in *C. elegans* *in vivo* . The biggest challenge is to ensure stable low levels of dCas9::NLS::2xEGFP::NLS protein in order to be able to distinguish distinct dCas9::NLS::2xEGFP::NLS foci from background fluorescence. Furthermore, it has previously been shown that high levels of *dCas9::EGFP* expression in mammalian tissue culture cells leads to the accumulation of dCas9::EGFP protein in nucleoli (Chen et al. 2014). With our inducible system, we can control the developmental stage and level of expression and therefore mostly prevent accumulation in nucleoli, especially during early embryonic stages. The CRISPR/dCas9 system is constantly being improved (Chaudhary et al. 2021) and the next challenge will be to target dCas9::NLS::2xEGFP::NLS to a non-repetitive genomic locus in *C. elegans* . The ultimate goal will be to use the CRISPR/Cas9 system to analyze chromatin dynamics *in vivo* by visualizing the opening (locus labeled by dCas9::NLS::2xEGFP::NLS) and closing of chromatin (locus non-labeled by dCas9::NLS::2xEGFP::NLS) in real time.

**Materials and Methods**

**Strains**

All strains were maintained and cultured at 15 ֯C as described (Brenner 1974). Mutations and transgenes used in this study are: LG III: *unc-119(ed3)* (Maduro et al. 1995). LG IV: *bcSi69* (this study) [P *hsp-16.41::dCas9::SV40-NLS::HA-Tag::GS-linker::SV40-NLS::GFP::GFP::SV40-NLS::mai-2 3’UTR* ]. Extrachromosomal arrays: *bcEx1367* (P *_U6_ rrn-4(sgRNAs1-4* ) targeting the *rrn-4* locus) (this study), *bcEx1368* (P *_U6_ rrn-1(sgRNAs1-4* ) targeting the *rrn-1* locus) (this study) and *bcEx1370* (P *_U6_ rrn-4(sgRNAs1-4* ) and P *_U6_ rrn-1(sgRNAs1-4* ) targeting the *rrn-1* and *rrn-4* loci) (this study). The single-copy integration allele *bcSi69* was generated using MosSCI (Frokjaer-Jensen et al. 2014). The strains used in this study are listed in below:

**Table:** 

**Strain**	**Genotype**	**Available from**
EG8081	*unc-119(ed3)* III; *oxTi177* IV	CGC
MD4195	*unc-119(ed3)* III *; bcSi69* IV	this study
MD4571	*unc-119(ed3)* III *; bcSi69* IV *; bcEx1367*	this study
MD4572	*unc-119(ed3)* III *; bcSi69* IV *; bcEx1368*	this study
MD4574	*unc-119(ed3)* III *; bcSi69* IV *; bcEx1370*	this study

**Plasmid constructions**

We mutated the RuvC1 site (D10A) and HNH site (H840A) in the *Sp* Cas9 gene derived from pDD162 (gift from Bob Goldstein (Addgene plasmid # 47549; Dickinson et al. 2013)) by site-directed mutagenesis using the following primers:

5’-CAGCATCGGCCTTGCCATCGGAACGAACTC-3’ and

5’-GAGTTCGTTCCGATGGCAAGGCCGATGCTG-3’ for mutating the D10A site;

5’-CTACGACGTCGACGCCATCGTCCCACAATC-3’ and

5’-GATTGTGGGACGATGGCGTCGACGTCGTAG-3’ for mutating the H840A site.

Both mutations lead to the inactivation of Cas9 (dCas9). Using Gibson cloning, we generated pBC1696 containing P *mai-2::dCas9::SV40_NLS::HA-TAG::GS-Linker::SV40-NLS::GFP::GFP::SV40-NLS::mai-2* 3’UTR in pCFJ305 (Frokjaer-Jensen et al. 2012). For better manipulation, we subcloned the entire insert of P *mai-2* :: *dCas9* ::SV40-NLS:: *HA-TAG* :: *GS-linker* :: *SV40-NLS* :: *GFP* :: *GFP* :: *SV40-NLS* :: *mai-2* 3’UTR into pBluescript II KS(+) to generate pBC1717. A second plasmid pBC1718 contained the *hsp16.41* promoter and the N-terminus of dCas9 in pBluescript II KS(+). By restriction digest, we exchanged the *mai-2* promoter with the *hsp-16.41* promoter. Specifically, we used SpeI and BstEII sites to cut out a 2,5 kb fragment containing *hsp16.41* and the N-terminus of dCas9 from pBC1718 to replace the *mai-2* promoter (3.7 kb) of pBC1717 to generate pBC1723. In this process, the dCas9 was fully reconstituted. The total insert (P *hsp16.41* :: *dCas9* :: *SV40-NLS* :: *HA-TAG* :: *GS-linker::SV40-NLS* :: *GFP* :: *GFP* :: *SV40NLS:* : *mai-2* 3’UTR) (9,5 kb) was subcloned into pCFJ350 using BssHII. This generated pBC1724.

**Table:** 

**Reagent**	**Description**	**available**
pBC1724	P *hsp16.41::dCas9::SV40-NLS::HA-TAG::GS-linker::SV40-NLS::GFP::GFP::SV40-NLS::mai-2* 3’UTR in pCFJ350	this study
pBC1723	P *hsp16.41::dCas9::SV40-NLS::HA-TAG::GS-linker::SV40-NLS::GFP::GFP::SV40-NLS::mai-2* 3’UTR in pBluescript II KS(+)	this study
pBC1718	the *hsp16.41* promoter and the N-terminus of dCas9 in pBluescript II KS(+)	this study
pBC1717	P *mai-2::dCas9::SV40-NLS::HA-TAG::GS-Linker::SV40-NLS::GFP::GFP::SV40-NLS::mai-2* 3’UTR in pBluescript II KS(+)	this study
pBC1696	P *mai-2::dCas9::SV40-NLS::HA-TAG::GS-Linker::SV40-NLS::GFP::GFP::SV40-NLS::mai-2* 3’UTR in pCFJ350	this study
pDD162	P *eft-3* ::Cas9 + Empty sgRNA	Addgene plasmid # 47549
pCFJ350	pCFJ350 - MCS( *ttTi5605* , II)	Addgene plasmid # 34866
pRB1017		Addgene plasmid # 59936
pJW1311		Addgene plasmid # 34866
pBluescript II KS(+)		Addgene

**sgRNAs**

All sgRNAs were designed by using the online sgRNA design tool https://cctop.cos.uni-heidelberg.de:8043 (Stemmer et al. 2015). The sgRNA oligos targeting *rrn-1* (LGI) or *rrn-4* (LGV) (see table below) were amplified as PCeU6::sgRNAs as shown previously (Ward 2015). As previously described, we generated PCR products, which were used for PCR-fusion to generate PCeU6::sgRNAs for each sgRNA separately (Ward 2015).

**Table:** 

Target gene	Sequence 5’à3’
*rrn-1* T1	CGAAAGTCTTTCGGTTCCGG
*rrn-1* T2	GATGCCATCTCGCGATTCGG
*rrn-1* T3	GCATGGCCGTTCTTAGTTGG
*rrn-1* T4	GGTTAAGAGGGACAAACCGG
*rrn-4* T1	GTTAGTACTTGGATCGGAGA
*rrn-4* T2	GCTTAACTTGCCAGATCGGA
*rrn-4* T3	ACTTGCCAGATCGGACGGGA
*rrn-4* T4	CGGAGACGGCCTGGGAATCC

**Microinjection**

Plasmids were purified using Qiagen’s Midi Plasmid Purification kit. The sgRNAs with the U6 promoter were amplified via PCR and then purified with the Qiagen Gel Extraction kit. Injection mixes were injected into the gonad arms of young adult hermaphrodite worms as described before (Mello et al. 1995). To generate MD4195, MosSCI injections were performed using strain EG8081. The plasmid pBC1724 was injected with a concentration of 15ng/ul. To generate the extrachromosomal arrays containing sgRNAs in the MD4195 background, we used pRF4 (150 ng/ul) as a co-injection marker. The sgRNAs were injected as PCR products ( *rrn-1* T1-T4 alone, *rrn-4* T1-T4 alone or *rrn-1* T1-4 + *rrn-4* T1-T4 together) as described before (Ward 2015) with a concentration of 25 ng/ul for each sgRNA.

***In vivo* imagining protocol**

**A. Strain maintenance**

All *C. elegans* strains were maintained at 15°C to avoid expression of the heat-shock inducible transgene *dCas9::NLS::2xEGFP::NLS* .

**B. Mounting embryos and imaging**

a. 10-20 gravid adults were dissected in water to release embryos. Embryos of the 12-20 cell stage were transferred to 2% agarose pads in water, and a cover-slip (22x22 #1.5) was added on top. Next, 20 µL of water was added under the cover-slip to prevent drying of the embryos, and the cover-slip was sealed using white vaseline.

b. To induce expression of the heat-inducible *dCas9::NLS::2xEGFP::NLS* transgene, the slide with the mounted embryos was subjected to heat-shock at 28°C (for MD4571, MD4574 and MD4195 [control strain]) or 29°C (for MD4572) in a thermocycler (lid-heating off) (SimpliAmp ^TM^ Thermocycler, Applied Biosystems) for 30 minutes.

(Caution: The lid-heating of the PCR machine should be switched-off or should be set to 28°C or less. Over-heating of the embryos will lead to over-expression of *dCas9::NLS::2xEGFP::NLS* , which can lead to accumulation of EGFP signal in the nucleoli.)

c. After the heat-shock, the embryos were incubated at 20 °C for 30 minutes for recovery before imaging.

d. Imaging was performed for 30-60 minutes using a 63X oil immersion objective on a Zeiss LSM 980 microscope with AiryScan2 detector. The details of the microscope settings are in tabular form below:

**Table:** 

Z-step size	0.17 µm
Excitation wavelength	488 nm
Laser power	3% (for MD4571, MD4574 and MD4195) 5% (for MD4572)
Pinhole	5.00 AU/ 394 µm
Effective Numerical Aperture	1.4
Detection wavelength	300-720 nm
Pixel time	0.65 µs
Frame time	1.12 s

**C. Image analysis: Nuclear foci counting**

a. The images were analyzed using Fiji (ImageJ Version 2.9.0). In order to count nuclear foci, a maximum intensity projection of the stack was generated.

b. Histogram matching: Once the maximum intensity projections for all embryos (including the control strain) were generated, the fluorescence intensity histograms were matched to ensure equalized intensity histograms for all embryos. (A caveat while using the LSM980 with AiryScan2 is that the fluorescent intensity histograms of different samples are different because of in-built optimization of fluorescent signal by the AiryScan detector. Therefore, it was important to match the fluorescent intensity histograms of the samples post image acquisition). In order to match histograms using Fiji (ImageJ), the maximum intensity projections of the various embryos were converted to stack, which leads to automatic histogram matching by Fiji (ImageJ). The histogram details for our samples after histogram matching are given below.

Min: 0

Max: 1925

Mean: 44.301

Standard deviation: 53.070

Bins: 256

Bin width: 7.520

c. Next, using the histogram matched maximum intensity projections, the nuclei in the embryo were identified and a region of interest was drawn around each nucleus.

d. The individual stacks of the maximum intensity projections of each nucleus were analyzed manually to count the number of foci in the nucleus (See Fig 1C, D, E).

e. The number of foci per nucleus was plotted as a percentage for each strain.

(Caution: Sometimes, two foci can be on top of each other and can be mistaken as a single focus. Therefore, it is important to look at the stack carefully.)

**Things to consider for troubleshooting**

1. Recovery time: The recovery time post heat-shock needs to be adjusted depending on the sgRNAs. For our experiments, a recovery time of 30 minutes was most suitable.

2. Embryo time-point for imaging: For our experiments, we used embryos at the ~50-200-cell stage as the expression levels were most consistent. In addition, we did not observe any background fluorescence in the control strain at this stage. However, in our experience, we noticed that in later stage embryos, dCas9::NLS::2xEGFP::NLS accumulation in intestinal cell nuclei can be observed, even in the control strain without sgRNAs.

3. Heat-shock temperature and timing: For this experiment, we performed heat-shock of the slide in a PCR machine at 28°C/29°C for 30 minutes with lid-heating switched off. It is important to note that not all PCR machines have the option to switch-off the lid-heating. In such cases, the lid should be kept open to avoid over-heating of the samples. In our experience, too much heat-shock leads to over-expression of *dCas9::NLS::2xEGFP::NLS* , and this can lead to EGFP accumulation in the nucleoli. Additionally, the heat-shock temperature and timings might need to be adjusted for different sgRNAs and different strains. Before collecting data, it is important to determine which heat-shock regime gives the best *dCas9::NLS::2xEGFP::NLS* expression and lowest background signal in the control strain without sgRNAs.
